# Register impacts perceptual consonance through roughness and sharpness

**DOI:** 10.3758/s13423-021-02033-5

**Published:** 2021-12-17

**Authors:** Tuomas Eerola, Imre Lahdelma

**Affiliations:** Department of Music, Durham University, Palace Green, DH1 3RL Durham, UK

**Keywords:** Consonance, Dissonance, Register, Roughness, Harmonicity, Perception

## Abstract

The perception of consonance and dissonance in intervals and chords is influenced by psychoacoustic and cultural factors. Past research has provided conflicting observations about the role of frequency in assessing musical consonance that may stem from comparisons of limited frequency bands without much theorizing or modeling. Here we examine the effect of register on perceptual consonance of chords. Based on two acoustic principles, we predict a decrease in consonance at low frequencies (roughness) and a decrease of consonance at high frequencies (sharpness). Due to these two separate principles, we hypothesize that frequency will have a curvilinear impact on consonance. A selection of tetrads varying in consonance were presented in seven registers spanning 30 to 2600 Hz. Fifty-five participants rated the stimuli in an online experiment. The effect of register on consonance ratings was clear and largely according to the predictions; The low registers impacted consonance negatively and the highest two registers also received significantly lower consonance ratings than the middle registers. The impact of register on consonance could be accurately described with a cubic relationship. Overall, the influence of roughness was more pronounced on consonance ratings than sharpness. Together, these findings clarify previous empirical efforts to model the effect of frequency on consonance through basic acoustic principles. They further suggest that a credible account of consonance and dissonance in music needs to incorporate register.

Musical consonance and dissonance (C/D) refers to the relative agreeableness/stability vs. disagreeableness/instability of simultaneous and successive pitch combinations. The topic has roots in antiquity and was studied by several 19th-century leading scholars such as Hermann von Helmholtz (1875) and Carl Stumpf (1898). Modern psychoacoustics has detailed how the sensory aspects of C/D relate to frequency (Terhardt, 1984) and to critical bands (Plomp and Levelt, 1965). The topic of C/D has received an increased amount of empirical attention recently (Friedman, Kowalewski, Vuvan, & Neill, 2021; Lahdelma & Eerola, 2020; Smit, Milne, Dean, & Weidemann, 2019) where more focus has been placed on the cultural aspects but also on the structure of the signal itself (such as the harmonicity or periodicity of the signal). In a large empirical review of all current explanations and computational models, Harrison and Pearce (2020) established convincingly that the acoustic and cultural components of C/D can be divided into three main constituents: *roughness*, *harmonicity* and *familiarity*. Roughness refers to the sound quality that arises from the beating of frequency components (Hutchinson & Knopoff, 1978), harmonicity indicates how closely a sonority’s spectrum corresponds to a harmonic series (Parncutt, 1989), and familiarity on both cultural and individual levels has been demonstrated to be an essential contributor to consonance perception (Lahdelma & Eerola, 2020; McLachlan, Marco, Light, & Wilson, 2013). The purpose of this study is to explore whether an important element has been omitted from recent C/D research, namely frequency, or hereafter *register* for clarity. Register’s possible role in the perception of C/D has been contemplated since the Middle Ages (see Tenney 1988), later the contribution of register to C/D has been captured in the form of *sharpness* (Zwicker & Fastl, 1990) denoting the high-frequency content of the stimuli.

When register has been directly manipulated, it has been observed to influence the perceived tension of intervals (Costa & Nese, 2020), melodic stimuli (Granot & Eitan, 2011), and valence and tension ratings of speech and music excerpts (Ilie & Thompson, 2006). The role of register has also been demonstrated to influence specifically C/D ratings when register has been manipulated (Lahdelma & Eerola, 2016). Similarly, register has impacted pleasantness ratings (as a proxy for consonance) when the mean pitch of the chords varied (Smit et al., 2019). When the register of the stimuli has been systematically varied between *G♯*_2_ (104 Hz) and *E*_4_ (659 Hz), musicians tend to rate theoretically consonant intervals (P5) as consonant also in the lower registers, while non-musicians do not show this tendency (Kung et al., 2014). Costa, Ricci Bitti, and Bonfiglioli (2000) found that harmonic intervals played in a low register (mean frequency 185.13 Hz) were perceived as more consonant compared to when they were played in a high register (mean frequency 1510.38 Hz). Later, Costa and Nese (2020) also observed that intervals and different types of noises played in a low register are perceived to be less tense, which could be interpreted as more consonant (cf., Lahdelma and Eerola (2020) than intervals in a *high* register, where *low* was defined as 130.8 Hz and above and high as 393.0 Hz and above. Contrasting findings were found by Granot and Eitan (2011) when their lower frequency range (73–139 Hz) was compared with the higher range (247–466 Hz), here the lower register produced higher tension ratings. Finally, McAdams and colleagues (2017) explored the affective qualities of instruments sounds—which included tension ratings—for real instrument sounds that spanned across the entire pitch register of the instruments. They found a U-shaped curve linking register and tension, where lower and higher register were perceived to be more tense. Drawing from all these observations, register may have a contribution to C/D perception, and it may operate differently in lower and higher frequencies due to different psychoacoustic factors (roughness vs. sharpness) affecting its perception.

Our research aim is to clarify how register impacts C/D ratings. More precisely, we assume that in the lower registers dissonance is increased due to more partials falling within the same critical band, which is wider relative to frequency within such registers (Moore & Glasberg, 1983; Zwicker & Fastl, 1990). In the highest registers, dissonance is assumed to be increased because of a high amount of sharpness (Lahdelma & Eerola, 2016, see Zwicker & Fastl, 1990). The aversive response to such high-frequency content is assumed to be related to the sensitivity of the human auditory system due to the amplification of resonances in the ear canal to frequency ranges above 1 kHz (Keefe, Bulen, Arehart, & Burns, 1993; McDermott, 2012). In sum, our pre-registered hypotheses were as follows (https://osf.io/76nhb/):

- *Hypothesis 1*: Register has a significant impact on C/D ratings. Both high and low registers deviate from the ratings given to chord stimuli in the middle register (roughly between *C*_4_ and *C*_5_).
- *Hypothesis 2*: Stimuli where the fundamentals are below 130 Hz (*C*_3_) dissonance will increase due to wider relative critical bandwidth at lower frequencies. For instance, at 100 Hz, the critical bandwidth is 95 Hz (95% in relative terms) whereas at 200 Hz the bandwidth is 104 Hz (52%), and at 400 Hz the bandwidth is 106 Hz (27%), at 800 Hz it is 145 Hz (18%) according to frequency to critical band (Bark) conversion equation (Traunmüller, 1990; Zwicker & Terhardt, 1980). Our predictions that stimuli below *C*_3_ will be perceived as dissonant stem from the fact that all fundamentals of a four-tone chord spanning an octave will be within the same critical band under this frequency.
- *Hypothesis 3*: When the fundamentals of the stimuli are above 1046 Hz (*C*_6_), they will be rated as more dissonant than their middle register (*C*_4_ to *C*_5_) counterparts due to increased sharpness.
- *Hypothesis 4*: Combining hypotheses 2 and 3, we predict an inverted U-shaped function for consonance and register (decreased consonance for the low and high registers), although the relative weight of these two separate elements may vary.
- *Hypothesis 5*: We predict that consonance ratings will also be impacted by the content of the stimuli and this will manifest itself as an *interaction* between *Chord type* and *Register*.

Our study design is a within-participant experiment, where we manipulate *Register* and *Chords*. We choose chords that vary systematically in their consonance and are balanced in terms of familiarity and roughness/harmonicity, which are the underlying elements of C/D to keep the stimulus set balanced. The plan was pre-registered prior data collection and the pre-registration can be found at https://osf.io/pj42b/.

## Methods

### Participants

Previous studies involving consonance ratings of piano chords have used between 15 (Popescu et al., 2019) to 40 participants (Lahdelma & Eerola, 2020) per group (an equal amount of musicians and non-musicians). In our study, we want sufficient power to detect predicted decreases in high and lower registers. We calculated the power required to detect differences between registers R1, R2, and R3 (and R5, R6, R7) by assuming the mean consonance ratings based on the theoretical predictors to be for R1 = 1.5, R2 = 2.75, R3 = 4.0, R4 = 4.0, R5 = 4.0, R6 = 2.75, R7 = 1.5. In our power size calculation, we also assumed homogeneous standard deviation of 1.20 and correlation of 0.80 between the conditions. Using simulation of power using the Superpower library (Lakens & Caldwell, 2021), we found that we need at least eight participants per condition (56 in total) to guarantee a power of 80% using the *p*= 0.01 level for the key comparisons in the low (R1 vs. R2, R2 vs. R3) or high registers (R5 vs. R6 or R6 vs. R7). In this design and sample size, we have ample power (100%) to detect the main effect of register.

### Stimuli

In the study by Lahdelma and Eerola (2020), the authors played 72 chords/intervals with piano timbre for 80 participants (40 non-musicians, and 40 musicians, Experiment 2 in the original study). To eliminate the possible confound of the number of pitches in the chords, we eliminate intervals as stimulus candidates and focus on chords with four pitches. From these chords, we chose four examples with a rationale that they are distinct in terms of roughness/harmonicity and familiarity and that they span each of the four quadrants of the roughness - familiarity space. Roughness, harmonicity and familiarity were extracted from symbolic data in the study and the dataset by Lahdelma and Eerola (2020), but it is worth noting that audio-based and symbolic-based roughness and harmonicity predictors correlate highly significantly (*r*> 0.70) when extracted from piano sounds in a large dataset of 4755 chords (Eerola & Lahdelma, 2021).

To determine this, we divide the stimulus space into four quadrants defined by low and high roughness/harmonicity and low and high familiarity. In this operation we split the stimulus based on a 50% quantile distribution of roughness values calculated from the symbolic information by the model by Hutchinson and Knopoff (1978) in the dataset provided by Lahdelma and Eerola (2020). A similar split is carried out with familiarity, where the familiarity model is based on a variant of a corpus model by Harrison (2020). We choose one chord from each quadrant defined by these two splits of the central variables for C/D in the past studies. We also check that we only include stimuli that would belong to the same quadrant based on *harmonicity* (using a model utilizing symbolic information by Stolzenburg (2015) to make sure there are no alternative predictions by harmonicity. Not all four quadrants will have chords that are represented as pitch classes with either three or four pitches, but each quadrant has chords that contain four separate pitches although in two specific cases, the chord contains a duplicated octave. We chose four chords from these quadrants, shown in Table 1, where we label the chords both by music-theoretic convention (Forte, 1973) and descriptive labels. The fundamental frequencies (*F*_0_s) of the pitches in each chord were adjusted so that the mean *F*_0_ of all pitches was always constant within a given register.

**Table 1:** Stimulus details

MIDI	Quadrant	Forte	Rating	Label
54 58 61 66	Low Rough. High Fam.	3-11B	10.000	Major triad
53 60 62 65	Low Rough. Low Fam.	3-7A	8.166	Power chord + M6
56 57 61 64	High Rough. High Fam.	4-20	6.033	Major 7th 3rd inv.
57 58 61 65	High Rough. Low Fam.	4-19A	2.194	Minor-Major 7th 3rd inv.

Quadrant refers to the calculated roughness and familiarity values (above or below 50% quantile for each predictor), Forte refers to the naming convention created by Allan Forte (1973) where each chord has a unique label based on the number of pitch classes and their respective order, rating refers to the consonance ratings by participants in Lahdelma and Eerola (2020), and label is the common music-theoretical description of the chord

**Fig. 1:**
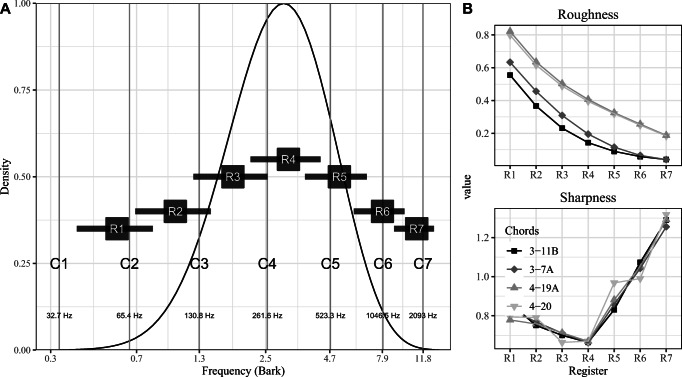
A 7 stimulus registers overlaid on an idealized distribution of orchestral instrument ranges (from Huron (2001), p. 8) shown as probability density. B Calculated roughness (Hutchinson and Knopoff (1978) model utilizing MIDI data) and sharpness values (calculated from audio using Zwicker’s 1990 model) for different chords across the stimulus registers

The four chords were generated in different registers using Ableton Live 9 implementing the *Synthogy Ivory Grand Pianos II* plug-in with *Steinway D Concert Grand* as the applied sound font. The registers will be defined by setting the lowest to mean pitch to 27 in MIDI (38.89 Hz) and generating six variants of the lowest register by successive transposition of ten semitones. This allows the chords to span the frequency range typically used by orchestral instruments where the highest pitch of the fundamental will be 100 in MIDI (2637.02 Hz). This spacing and the choice of the lowest and highest frequency is motivated by the analysis of the complete register of several orchestral instruments reported by Huron (2001) (p. 8) and provides some overlap between the lowest and highest pitches of the chords between registers. The frequency ranges of the stimuli across the registers are shown in Fig. 1 panel A and the impact on roughness and sharpness, as measured with models by Hutchinson and Knopoff (1978) (from MIDI) and Zwicker and Fastl (1990) (from audio), respectively, are shown in panel B. The former model was implemented through the incon package for R (Harrison & Pearce, 2019) relying on symbolic information and the latter from audio through Matlab scripts created by Claire Churchill, which replicates Zwicker’s model of sharpness (Zwicker & Fastl, 1990).

Panel B in Fig. 1 displays a dramatic increase in roughness provided by Hutchinson’s model in the lower registers (particularly registers 1 and 2). Also, sharpness increases linearly in registers 5, 6, and 7, which we predict will translate into lower consonance ratings for all chords.

### Procedure

We collected the data using an online platform, PsyToolkit (Stoet, 2017) and recruited the participants from prolific.ac.uk. The participants had to use headphones to participate and we implemented a headphone check created by Woods, Siegel, Traer, and McDermott (2017). Our main variable consists of the consonance ratings which are self-reports carried out using a scale from 0 (dissonant) to 10 (consonant). We replicate the instructions and the rating procedure from Lahdelma and Eerola (2020) with the exceptions of collecting the data using a visual slider and an underlying continuum of dissonance to consonance from 0 to 10. For auxiliary analyses, we used an index of musical expertise as defined by a 1-item OMSI question (for the benefits of using this strategy to assess musical expertise, see Zhang and Schubert (2019)) and descriptors for the education level, gender, and age of the participants.

### Data analysis

The main analysis will apply linear mixed models to estimate the effect of the main manipulated factors, *Register* (7 levels) and *Chord* (4 levels) on consonance ratings with *Participant* as a random factor. We use the lmer program of the lme4 package (Bates, Sarkar, Bates, & Matrix, 2007) for estimating fixed and random coefficients within R programming environment (R Core Team, 2020). The analysis scripts and full data are available at GitHub https://github.com/tuomaseerola/consonance-register.

## Results

Out of 77 recruited participants, ten failed the headphone check, seven failed to complete the task, one failed the consistency check (mean absolute error of ≥ 4 in three repeated items), and four scored negative correlations with the scores of the mean of other participants. These criteria were defined in the pre-registration plan. This left 55 participants in total. To check the validity of our stimulus choices, see auxiliary analysis 1.

To identify the differences in ratings between different levels of Register and Chord, we coded Register as a linear predictor (+ 1 to + 7) and Chord as a linear factor (+ 1 to + 4 in the order of consonance as arranged in Table 1). A linear mixed model analysis yielded a significant main effect of Register (*t*(1587) = 12.72, *p* < .001) but the linear effect of Chord (*t* = -1.15, *p* = .248) was not significant. The interaction between Chord and Register was significant at the *p*<.001 level (see Table 2 for details). These results match our hypotheses well (H1 and H5 are supported), although this initial analysis did not address the shape of the relationship between consonance and register (H2 to H4).

**Table 2:** Linear mixed model analysis results for the consonance ratings for two factors

Term	$\hat {\beta }$	95% CI	*t*	*df*	*p*
Intercept	2.92	[2.22, 3.61]	8.21	1,278.58	<.001
Chord	-0.14	[-0.38, 0.10]	-1.15	1,590.00	.248
Register	0.96	[0.81, 1.10]	12.72	1,590.00	<.001
Chord × Register	-0.16	[-0.21, -0.10]	-5.72	1,590.00	<.001

To investigate how Register impacted consonance ratings, we carried out a post hoc analyses—adjusted for multiple comparisons with Tukey’s method—of Register which yielded significant differences between all pairings except Register 4-5, 4-6, 4-7, 5-6, 6-7 at the level of *p*<.001. To test the specific hypotheses about lower and higher registers receiving lower consonance ratings due to the acoustic effects, we first tested the hypothesis 2 by applying a contrast between the lowest two registers (R1 and R2) and the other registers (R3-R7). The mean of the lowest two registers was significantly lower (M = 2.91, SE= 0.355) than the consonance ratings for the rest of the registers (M = 5.58, SE= 0.380), *df* = 194, *t*= 36.330, *p*<.0001. According to hypothesis 3, the two highest registers (R6 and R7) should have decreased consonance compared to the middle register (R4-R5), and this hypothesis is supported by the differences in the means (M = 5.96, SE= 0.372 and M = 5.71, SE= 0.378 for R6-R7 and R4-R5 (*t*= 2.225, *p*= 0.0263), albeit this difference is more modest than the one exhibited by the comparison of lowest registers to the rest of the registers.

**Fig. 2:**
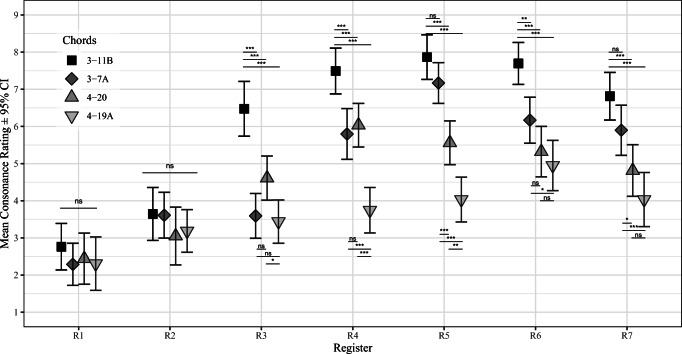
Consonance ratings across Chord and Register

We followed-up the interaction between Chord and Register from the LMM analysis with post hoc tests for the differences across the four Chords in different Registers using Tukey’s correction for multiple testing. The exact test results are displayed in Fig. 2. The overall pattern of results suggests that the four chords differed in a predictable way in the middle register (3-11B is the most consonant, the 3-7A and the 4-20 chords share the middle consonance position in the middle register (R4), and the 4-19A chord is the least consonant). However, although the ranked order of the consonances/dissonances of the chords remain largely similar across register, there is considerable variation in the differences between the consonance ratings at different registers; In the lowest two registers (R1-R2) all chords were rated as dissonant and there are no statistically significant differences between the chords. In the R3 register, all other chords except the major chord (3-11B) have been rated as dissonant and the differences between the other chords is compressed. Above the middle register (R4), the differences between most of the chords remain statistically significant although the mean of all chords drop in the highest register.

After these theory-driven predictions about specific registers and interactions between Chord and Register, we tested whether the impact of Register on consonance is best characterized by an inverted U that captures both decreases in consonance due to acoustic interference in the lower registers and increased sharpness in the highest registers. This comparison was carried out in context of a linear mixed model using Register as a fixed factor whilst holding the participant ID as the random factor. A quadratic relationship between consonance and register is better than a linear function (*χ*^2^= 103.75, *p*<.001, marginal *RGLMM*2 = 0.139 and AIC= 8021 for the linear model, *RGLMM*2 = 0.187, AIC= 7926 for the quadratic model). Using the same analytical setup, a cubic function provides a better fit than the quadratic model (*RGLMM*2 = 0.191, AIC= 7924, *χ*^2^= 10.77, *p*<.01), albeit the increase is rather marginal. The added 0.4% of variance explained by the marginal *RGLMM*2 is rather small considering the added complexity brought in by a third parameter, but nevertheless, a cubic function offers a better account of the consonance ratings even when this added complexity is taken into account through Akaike’s Information Criterion (AIC), which shows the lowest values for the cubic function.

**Fig. 3:**
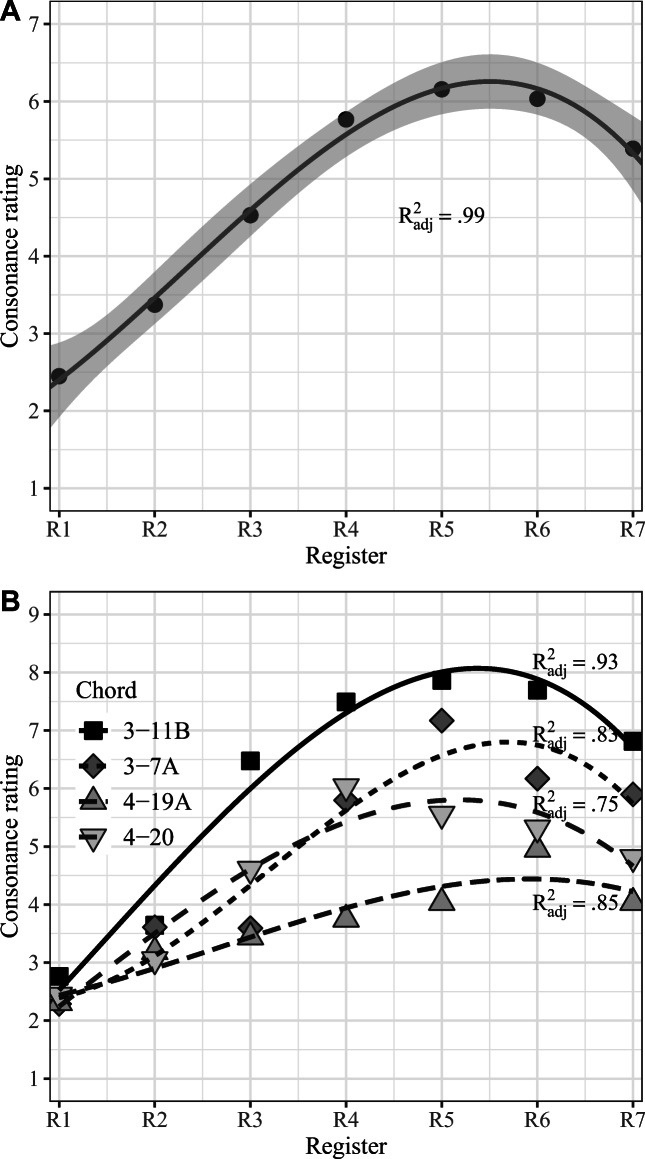
Cubic model fit to (A) mean consonance ratings and (B) means across the chords

To summarize this finding, we collapsed the consonance ratings to means across register and chords and applied the best fitting model (cubic) to this data, shown in Fig. 3. Even with the means across register only (panel A), the cubic model (*R*^2^=.99, 90% CI [0.90, 1.00], *F*(3,3)= 166.32, *p*=.001) is better than the quadratic model (*R*^2^=.97, 90% CI [0.75, 0.99], *F*(2,4)= 63.32, *p*=.001), which is better than the linear model (*R*^2^=.72, 90% CI [0.15, 0.92], *F*(1,5)= 13.07, *p*=.015). These analyses provide support for hypothesis 4, which assumed an inverted U-relationship between consonance and register due to two different psychoacoustic explanations. What we did not specify in our hypothesis was which explanation would be able to impact the consonance ratings more strongly, critical bandwidth in the low register or sharpness in the high register. The shape of the curve and the means across the registers suggest that consonance ratings in the low registers (R1-R2) were more impacted by psychoacoustics than the high registers (R6-R7), also supported by a significant contrast between R1-R2 and R6-R7 (*df* = 1539, *t*= 17.28, *p*<.0001).

Finally, we evaluated to what degree the two key acoustic models account for the consonance ratings using regression with the means across chords and registers. With a linear combination of roughness and sharpness, 78% of the variance in consonance ratings could be accounted for (*R*^2^=.78, 90% [*CI*0.59 0.87], *F*(2,25)= 44.67, *p* <.001), where roughness (*β*=-7.52, CI 90% -9.22 -5.82, *t*=-9.11, *p*<.001) and sharpness (*β*=-2.17, CI 90% -4.01 -0.32, *t*=-2.42, *p*=.023) operated the way predicted (significant contribution to the ratings and negative coefficients). Slightly better overall fit (*R*^2^=.84) can be achieved with polynomial variant of roughness. In the linear model, roughness seems to be the predictor that explains most of the variance, measured by semi-partial correlation (*sr*=-.852, *p* <.001) between roughness and consonance with sharpness partialled out. For sharpness, the semi-partial correlation is notably lower and non-significant (*sr*=-.226, *p*=.257) when roughness has been partialled out. In effect, roughness explains *R*^2^=.726 whereas sharpness only accounts for *R*^2^=.066 of the consonance ratings. For additional exploratory analyses about musical expertise, see auxiliary analysis 2.

## Discussion

We found that consonance ratings of chords to be heavily influenced by the frequency range in which the chords are presented. The potential effect of register on consonance ratings has been hinted at in past research (Costa & Nese, 2020; Granot & Eitan, 2011; Lahdelma & Eerola, 2016; Smit et al., 2019; Zwicker & Fastl, 1990) although the results concerning consonance or the closely related concept of tension have been conflicting, and the full range of register has not been explored previously. Based on two separate psychoacoustic effects with concomitant models (roughness and sharpness), we predicted specific decreases in consonance for both low and high registers. The findings were consistent with the predictions and the non-linear relationship between register and consonance could be accurately described with a cubic model. The two acoustic predictors adequately characterized this pattern of observations, although roughness accounted for the dominant part of the overall variance in consonance ratings. The results are also consistent with recent findings about the central role of the critical bandwidth in perceived dissonance; Armitage, Lahdelma, and Eerola (2021) demonstrated that automatic responses to the dissonance of intervals influence affective priming specifically when the fundamentals of the intervals are within the critical bandwidth.

The findings are limited to a single timbre, albeit the most common one (piano) utilized in most C/D studies (see Lahdelma & Eerola 2020). Different timbres will behave differently across the register as the magnitude of the partials will interact with critical bandwidth at the low frequencies and may pronounce the effect of sharpness at high frequencies. The present study lays the foundation of what would be expected with different timbres. It is also worth mentioning that sharpness is an aspect of timbre that is a complex, multidimensional phenomenon, and there are multiple ways preferences for sounds may be impacted by timbral properties, including sharpness (Herbst, 2019), spectral crest, and spectral centroid (McAdams, Douglas, & Vempala, 2017).

The results are consistent with the advice on voicing chords in music; so called close position voicing (where the tones of the chords are in a compact form and within an octave) is to be avoided in low register as they “sound muddy” (McGowan, 2011; Plomp & Levelt, 1965). We surmise that violations of this fundamental music theoretical principle in the low register contributes to the lower consonance ratings of musicians in comparison to non-musicians. Most orchestration guides also warn of arranging instruments and chords to high registers as they will sound “shrill” or “harsh” (Adler, 2002; McGowan, 2011).

The implication of the results is that assessment and modeling of C/D needs to take register into account. A recent review of C/D models (Harrison & Pearce, 2020) did not include this aspect arguably due to the narrow range of register in past datasets. The current study and the open data offer good prospects to further improve the modeling of C/D. A recent study (Eerola & Lahdelma, 2021) suggests that sharpness has an impact on C/D although the impact of sharpness was small in comparison to roughness in their study and also in the present study. As such, it can be concluded that sharpness has a limited yet distinct impact on perceived C/D as the effect is confined to exclusively the highest register. Overall, this study has demonstrated that a credible account of consonance and dissonance in music needs to incorporate the aspect of register.

## Data Availability

All code is available at https://github.com/tuomaseerola/consonance-register

## Appendix

### Auxiliary Analysis 1: Consonance and Stimulus Design

Our four chords were selected based on past consonance rating data (Lahdelma & Eerola, 2020) and acoustical controls, where we selected chords with either low or high levels of roughness (Hutchinson & Knopoff, 1978) and familiarity (Harrison, 2020) and having clear differences in consonance ratings. As an auxiliary analyses, we carried out linear mixed model analysis with Roughness and Familiarity as the fixed factors and participants as a random factor. This analysis showed a significant main effect of Familiarity (*t*= 4.53, *p*<.001) and Roughness (*t*= 8.37, *p*<.001) but no significant interaction between the Factors (*t*= 1.24, *p*=.22). This confirms our basic assumption that the chord differs in consonance across familiarity levels (high familiarity M = 5.34, SD= 3.10, low M = 4.31, SD= 2.81) and roughness (high roughness M = 4.11, SD= 2.77, low M = 5.54, SD= 3.09). The lack of interaction is also consistent with the design principle and the past research that has suggested that these two aspects of C/D are relatively unconnected (Eerola & Lahdelma, 2021; Harrison & Pearce, 2020).

### Auxiliary Analysis 2: Consonance and Musical Expertise

As an exploratory analysis, we checked whether musical expertise impacted the consonance ratings and interacted with the chords and register. We had collected information about expertise using the 1-item OMSI questionnaire (Zhang & Schubert, 2019). From this question with 6 categories, we coded all non-musicians except those who reported themselves as “amateur musician,” “serious amateur musician,” “semi-professional musician,” or “professional musician.” Altogether 16 participants qualified as musicians and 44 as non-musicians. We added this categorical factor into the LMM analyses involving Chord and Register. This did not yield significant effect of Musical Expertise (*t* = 0.39, *p* = .694) nor were there any significant interaction with Chord (*t* = 0.24, *p* = .809) or Register (*t* = 1.23, *p* = .219).
